# NKG2A and circulating extracellular vesicles are key regulators of natural killer cell activity in prostate cancer after prostatectomy

**DOI:** 10.1002/1878-0261.13422

**Published:** 2023-03-27

**Authors:** Yu‐Chuan Lu, Chen‐Hsun Ho, Jian‐Hua Hong, Ming‐Chieh Kuo, Yi‐An Liao, Fu‐Shan Jaw, Jason Chia‐Hsien Cheng, Chao‐Yuan Huang, Ko‐Ping Chang, Chung‐Hsin Chen, Jung‐An Lin, An Hsiao, Hsiu‐Ni Kung

**Affiliations:** ^1^ Department of Surgical Oncology, National Taiwan University Cancer Center National Taiwan University College of Medicine Taipei Taiwan; ^2^ Department of Urology National Taiwan University Hospital Taipei Taiwan; ^3^ Division of Urology, Department of Surgery Shin Kong Wu Ho‐Su Memorial Hospital Taipei Taiwan; ^4^ School of Medicine, College of Medicine Fu Jen Catholic University New Taipei City Taiwan; ^5^ Institute of Biomedical Engineering National Taiwan University Taipei Taiwan; ^6^ Department of Urology National Taiwan University Hospital, Yunlin Branch, National Taiwan University College of Medicine Taipei Taiwan; ^7^ Division of Radiation Oncology, Department of Oncology National Taiwan University Hospital Taipei Taiwan; ^8^ Graduate Institute of Oncology National Taiwan University College of Medicine Taipei Taiwan; ^9^ Department of Pathology National Taiwan University Hospital Taipei Taiwan; ^10^ Graduate Institute of Anatomy and Cell Biology, College of Medicine National Taiwan University Taipei Taiwan

**Keywords:** circulating EVs, ligand alterations, natural killer cell activity, NKG2A, prostate cancer, robotic‐assisted radical prostatectomy

## Abstract

Extracellular vesicles (EVs) are an important regulatory factor for natural killer cell activity (NKA) in the tumor microenvironment. The relationship between circulating EVs in the peripheral blood and natural killer (NK) cells in prostate cancer (PCa) is unclear. This study aimed at investigating the key regulators in the interaction between circulating EVs and NK cells in PCa patients before and after tumor removal. NK‐cell characteristics were prospectively assessed in 79 patients treated with robot‐assisted laparoscopic radical prostatectomy preoperatively and postoperatively. Compared with healthy donors, the existence of prostate tumors increased the number of circulating EVs and altered ligand expression of EVs. Circulating EVs extracted from cancer patients significantly decreased NKA of NK cells compared with those extracted from healthy donors. Upon treatment with an inhibiting antibody or small interfering RNA, natural killer cell protein group 2A (NKG2A) was identified as the main NKA regulator in cancer patients for accepting the signal from circulating EVs. After surgery, NKA was increased and NKG2A expression on NK cells was significantly reduced. The expression of ligands for natural killer cell protein group 2D (NKG2D) on EVs and the level of circulation EVs both significantly increased. With the decrease in NKG2A levels on NK cells and the increase in total NKG2D ligands on circulating EVs, which was increased postoperatively, both NKG2A on NK cells and NKG2D ligands on circulating exosomes are main regulators of NKA restoration after prostatectomy.

## Graphical abstract

More circulating extracellular vesicles (EVs) and downregulated natural killer cell activity (NKA) were found in prostate tumor patients. The inhibiting receptor, natural killer cell protein group 2A (NKG2A), was shown to be the main receptor regulating NKA through EV binding. After prostatectomy, the number of circulating EVs increased, which subsequently increased NKA by decreasing NKG2A on NK cells and increasing total NKG2D (activating receptor for NKA) ligands on EVs.

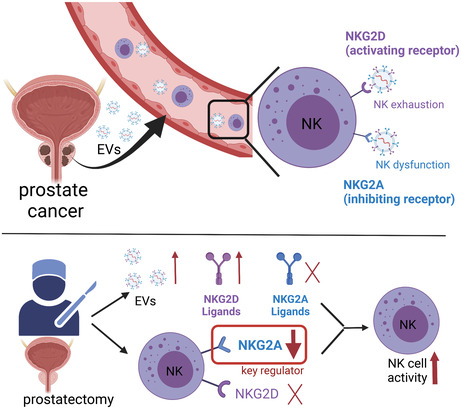

**Abbreviations**
BCA: bicinchoninic acid
DiI: 1,1′‐dioctadecyl‐3,3,3′,3′‐tetramethylindocarbocyanine perchlorate
DMEM: Dulbecco's modified Eagle medium
EV: extracellular vesicle
FI: fluorescence intensity
HLAE: human leukocyte antigen E
IFNr: interferon production regulator
IFN‐γ: interferon‐γ
IHC: immunohistochemistry
IQR: interquartile range
MICA1: MHC class I polypeptide‐related sequence A1
MTT: 3‐(4,5‐dimethylthiazol‐2‐yl)‐2,5‐diphenyl tetrazolium bromide)
NK: natural killer cell
NKA: natural killer cell activity
NKG2A: NK‐cell lectin‐like receptor subfamily C member 1/natural killer cell protein group 2A
NKG2D: NK‐cell lectin‐like receptor subfamily K member 1/natural killer cell protein group 2D
NTA: nanoparticle tracking analysis
PBMC: peripheral blood mononuclear cell
PCa: prostate cancer
PEG: polyethylene glycol
PS: penicillin–streptomycin
RARP: robot‐assisted laparoscopic radical prostatectomy
RT: room temperature
SD: standard deviations
SEC: size exclusion chromatography
siRNA: small interfering RNA
TEM: transmission electron microscopy
ULBP1: UL16 binding protein 1

## 1 Introduction

The immune system is crucial for human health, and targeting immune checkpoints in cancer therapy has been investigated recently in an increasing number of studies [1, 2]. Most current treatment options that control the tumor microenvironment focus on T‐cell immunity by promoting activation or by suppressing inhibitory signals. The limited success achieved by T‐cell immunotherapy highlights the importance of developing new‐generation immunotherapeutics, such as the use of previously ignored natural killer (NK) cells [3]. NK cells are the main effector cell type in innate immunity in the peripheral blood and intratumor regions, and these cells are capable of killing tumor cells and virus‐infected cells at a very early stage via cytotoxic activity and cytokine production in the immune system [4]. NK cells influence the adaptive immune system by secreting proinflammatory cytokines, such as interferon‐γ (IFN‐γ), to counteract the escape mechanisms promoted by tumor cells [5] and accelerate the eradication of malignant cells [6].

Liquid biopsy was recently suggested as a better method to check patient status than the more invasive tumor biopsy [7]. Extracellular vesicles (EVs) are cell‐derived nanosized (30–150 nm in diameter) lipid bilayer‐encapsulated vesicles that are found in most biological fluids, including blood. EVs in the tumor microenvironment impair NK‐cell function by inducing NK‐cell dysfunction or exhaustion [8]. Most normal and cancer cells produce EVs. Although tumor‐derived EVs serve as tumor biomarkers [9], the combination of EVs in the peripheral blood provides a snapshot of the entire tumor environment. Therefore, the total circulating EV burden may identify disease [10]. However, how the levels of total circulating EVs are altered in prostate cancer (PCa) before and after the removal of the prostate tumor or the characteristic alterations of EVs are not known. To solve this problem, a cohort of PCa patients from the National Taiwan University Hospital was established.

Activating and inhibiting NK‐cell receptors controls NK‐cell activity (NKA) [11]. Due to the limited production of receptors for distinguishing incalculable numbers of potential antigens specifically, NK cells identify target cells by maintaining a precise balance between activating costimulatory and inhibitory signals. These signals fine‐tune decisions on the activation and functional status of NK cells. Natural killer cell protein group 2D (NKG2D) and NKG2A are two major receptors that were investigated previously. Decreased NKG2D expression levels are observed in most cancer patients [12, 13, 14, 15]. A reduction in NKG2D expression levels correlates with decreased NK‐cell function with disease progression [16, 17, 18]. In contrast, NKG2A expression levels were higher in NK cells infiltrating breast tumors compared with cells isolated from symmetric normal breast tissue [12] and in peripheral NK cells of patients with acute myeloid leukemia compared with NK cells of age‐matched controls [19]. The interaction of receptors on NK cells and ligands on EVs regulates NKA [20]. Although the interaction between ligands on EVs and receptors on NK cells may be the main regulator of NKA, there is limited knowledge about alterations in EVs, including their number and receptor expression on the membrane, after the surgical removal of PCa. The present study investigated the regulatory mechanisms between EVs and NKA at the cellular level before and after prostatectomy.

## 2 Materials and methods

### 2.1 Patient recruitment and data collection

This prospective observational cohort study was approved by The Institutional Review Board and Ethics Committee of National Taiwan University Hospital (IRB 200903039R and IRB201711106RINC). All study methods were executed with the corresponding guidelines and regulations of the National Taiwan University Hospital. The study methodologies conformed to the standards set by the Declaration of Helsinki. The experiments were undertaken with the understanding and written consent of each subject. All study participants provided informed consent. From March 2017 to July 2019, 83 men with biopsy‐proven PCa who underwent robot‐assisted laparoscopic radical prostatectomy (RARP) were prospectively enrolled from the National Taiwan University Hospital. The following eligibility criteria were used for patient enrollment: (a) no history of diagnosis or treatment for other malignancies; (b) no history of neoadjuvant androgen deprivation therapy for PCa; (c) no history of inflammatory conditions as assessed by a white blood cell count < 10 000 cells·mL^−1^ and C‐reactive protein level < 1.0 mg·L^−1^; and (d) no history of exposure to immunosuppressive agents. All the methods were followed in the previous literature [21]. A total of 79 patients were ultimately enrolled in this study.

Blood samples were collected before and 4–6 weeks after RARP in Vacutainer green lithium‐heparin tubes (BD Biosciences, San Jose, CA, USA). The clinicopathological profiles of the participants are listed in Table S1. To define a standard for comparing the proportional change in NK‐cell number in peripheral blood mononuclear cells (PBMCs), blood from a healthy male volunteer was collected at each time point as a reference, and 13 healthy volunteers who donated blood were included in this study. The ages of healthy donors ranged from 30 to 78 years old.

### 2.2 Cancer stage classification

Prostate cancer staging was performed in accordance with the 8th American Joint Committee on Cancer (AJCC) Tumor, Node, Metastasis (TNM) system guidelines. The pretreatment risk stratification was determined based on National Comprehensive Cancer Network (NCCN) guidelines, and tumors were graded using the Gleason score consistent with International Society of Urological Pathology (ISUP) guidelines. Postoperative progression was defined as biochemical failure according to National Comprehensive Cancer Network criteria or as a secondary PCa treatment, including pelvic radiation or androgen deprivation therapy.

### 2.3 NK‐cell fraction

Peripheral blood mononuclear cells were isolated from blood using Lymphoprep™ (density, 1.077 ± 0.001 g·mL^−1^; Blossom Biotechnology, Taipei, Taiwan). Briefly, 2 mL of blood was mixed with the same amount of PBS and carefully loaded on the top of 4 mL of Lymphoprep in a centrifuge tube. After 20 min of centrifugation at 800 ***g***, the middle white layer was obtained, and PBMCs were isolated after two PBS washes. PBMCs were stained with CD3, CD56, and CD16 (CD3‐BUV395 (UCHT1), CD16‐BV421 (3G8), CD56‐APC (B159) BD Horizon Brilliant™ (BD Bioscience)) for 30 min at 37 °C. CD3^−^/CD56^+^/CD16^+^ cells were considered NK cells, and NK‐cell fraction was calculated using BD LSRFortessa™ flow cytometry (BD Bioscience). The gating strategy for NK cells is shown in Fig. S1A. For comparison in each flow cytometry analysis, one tube of blood from a healthy male volunteer was processed using the same protocol each time and used as the normal control. A total of 13 healthy donors participated in the study.

### 2.4 Receptors on NK cells

Peripheral blood mononuclear cells were stained with antibodies targeting receptors, including NKG2D (NKG2D‐BB515 (1D11)) and NKG2A (NKG2A‐PE (131411)), for 30 min at 37 °C. The fluorescence intensities (FIs) of NKG2D and NKG2A on CD3^−^/CD56^+^/CD16^+^ NK cells were quantitated using a Fortessa flow cytometer.

### 2.5 Natural killer cell activity

Natural killer cell activity was measured using NKVue (ATGen, Seongnam, South Korea) [21, 22]. Briefly, 1 mL of blood was added to the reaction tube containing PROMOCA™, which specifically stimulates the release of IFN‐γ from NK cells. The tube was incubated at 37 °C for 24 h and centrifuged at 600 ***g*** for 15 min. The supernatants were harvested for IFN‐γ ELISA. Samples with test results > 2000 pg·mL^−1^ were reanalyzed at a 1 : 10 dilution.

### 2.6 EV extraction and analysis

Patient sera were separated from blood via centrifugation and stored at −80 °C until use following the procedure [23]. EVs were separated from serum using the Exoquick Exosome Extraction Kit (System Biosciences, Palo Alto, CA, USA). Briefly, 250 μL of serum was mixed with 63 μL of Exoquick and incubated at 4 °C for 30 min. EVs were isolated after centrifugation at 1500 ***g*** for 30 min, suspended in 500 μL PBS, and stored at −20 °C for subsequent experiments. EV morphology was observed under transmission electron microscopy (TEM; Hitachi, Tokyo, Japan) according to a previously described method [24]. EV number and size were measured using nanoparticle tracking analysis (NTA; Malvern Panalytical, Malvern, UK). EV protein concentration was measured by BCA (bicinchoninic acid) protein assay kit (T‐Pro Biotechnology, Zhonghe, Taiwan).

### 2.7 Markers and ligands on EVs

Equal volumes of EV solution were mixed with sample buffer. Proteins were separated in a 10% SDS‐acrylamide gel and transferred to nitrocellulose membranes (BioTrace™ NT Nitrocellulose Transfer Membrane; PALL, Washington, NY, USA). CD63, MHC class I polypeptide‐related sequence A1 (MICA1), UL16 binding protein 1 (ULBP1), and human leukocyte antigen E (HLAE) were targeted with primary antibodies (1 : 1000; AB62540, AB176566, AB2216, and AB124783, respectively; Abcam, Cambridge, MA, USA) and corresponding secondary antibodies. CD63, an endosome‐specific tetraspanin, is a recognized EV marker [25]. Protein images were observed using a UVP bioimaging system (UVP GelStudio Plus; Analytik Jena, Jena, Germany) with enhanced chemiluminescence (LumiLong Plus chemiluminescence detection kit, T‐Pro Biotechnology). Protein levels were analyzed using imagej software (National Institutes of Health, Bethesda, MD, USA).

### 2.8 The interaction between EVs and NK cells

Extracellular vesicles were stained with CellMask™ deep red plasma membrane stain (5 μg·mL^−1^; Thermo Fisher Scientific, Auburn, AL, USA) at 37 °C for 30 min. After washing with PBS, the stained EVs were incubated with NK cells for 24 h. The nuclei of NK cells were labeled with Hoechst (1 : 20 000). Deep red staining of EVs was observed under fluorescence microscopy (Leica, Wetzlar, Germany). Cells were collected, and the intensity of deep red fluorescence was measured using flow cytometry (Calibur; BD).

### 2.9 NK‐cell cytotoxicity

The NK cytotoxicity assay was performed using K562 leukemia cells as target cells as previously described with some modifications [26]. NK92‐MI cells (RRID: CVCL_3755), which were purchased from ATCC (Manassas, VA, USA), were cultured in α‐minimum essential medium supplemented with inositol (0.2 mm), folic acid (0.02 mm), β‐mercaptoethanol (0.1 mm), 12.5% FBS; (Gibco/Thermo Fisher Scientific), and 12.5% horse serum (Gibco, Thermo Fisher Scientific). Forty microgram protein per 5 μL of EVs were added to 1 × 10^6^ NK cells and incubated for 24 h. K562 cells (RRID: CVCL_0004), which were purchased from BCRC (Hsinchu, Taiwan), were cultured in Dulbecco's modified Eagle medium (DMEM) with 10% FBS and 1% penicillin–streptomycin (PS; Gibco), labeled with 1,1′‐dioctadecyl‐3,3,3′,3′‐tetramethylindocarbocyanine perchlorate (DiI; Thermo Fisher) at 4 °C for 15 min and washed with PBS to remove excess DiI. DiI‐labeled K562 cells were added to NK cells at a 2 : 1 ratio and incubated for 2 h. Two sets of controls were prepared: NK92 cells with DiI‐labeled K562 cells for 2 and 0 h without EVs. The FI between these two sets of controls was set as 100% cytotoxicity. For the neutralization of receptors on NK cells or the ligands on EVs, neutralizing antibodies targeting NKG2D, NKG2A, and HLAE were incubated with NK cells or EVs for 2 h, and the subsequent NK cytotoxicity was assessed as described above. Both cell lines used in the study have been confirmed by flow cytometry with marker labeling (NK92‐MI: CD56^+^/CD3^−^, K562: CD45^+^/CD146^+^) at the beginning of experiments, and all experiments were performed with mycoplasma‐free cells.

### 2.10 Small interfering RNA transfection

Small interfering RNAs (siRNAs; 50 ng) targeting nothing (negative control), NKG2A, and NKG2D (smart pools from Synbio Tech, Kaohsiung, Taiwan) were incubated with 3 μL polyfast transfection reagent (MedChemExpress, Princeton, NJ, USA) in medium without FBS for 15 min. Then, the mixture was added to medium containing 5 × 10^4^ NK cells and incubated for 48 h. Forty microgram protein per 5 μL of circulating EVs from healthy donors and tumor patients were added to NK cells for 24 h. A total of 1 × 10^5^ DiI‐labeled K562 cells were added to NK cells for 2 h, and the remaining DiI‐labeled K562 cells were measured by flow cytometry with an FL2‐H filter.

### 2.11 Cell survival assay

DU145 PCa cells were maintained in DMEM with 10% FBS and 1% PS. 1 × 10^4^ DU145 cells were seeded in the 48‐well plate, and treated with EVs (20 μg)‐treated NK92 cells (5 × 10^4^) for 24 h. 0.5 mg·mL^−1^ of MTT [3‐(4,5‐dimethylthiazol‐2‐yl)‐2,5‐diphenyl tetrazolium bromide] was added to each well, and then the plates were incubated at 37 °C for 3 h. The MTT was then removed, the formazan product was dissolved in 100 μL of DMSO for 10 min, and the absorbance at 550 nm was measured using a microplate reader. The optical density value of DU145 treated with pre‐EV was set as 100% cell survival in each pair of pre‐post EVs. Six sets of EVs from both stage 2 and stage 3 were used in the cell survival assay.

### 2.12 Immunohistochemistry

Slides with PCa paraffin sections were dewaxed and rehydrated with xylene and sequential concentrations of alcohol from 100% to 75%. After a 5‐min wash with tap water, antigen retrieval was performed with 0.1 m sodium citrate buffer with 0.5% Tween 20 for 20 min in a microwave. The samples were blocked with blocking solution (10% serum with 0.1% Triton X‐100) for 1 h at room temperature (RT) and incubated overnight with primary antibodies (HLAE and PE‐conjugated NKG2A, 1 : 200) at 4 °C. After five washes with PBS, the samples were incubated with Alexa 488‐conjugated secondary antibody for HLAE (1 : 500) for 1 h at RT. After 10 min of Hoechst labeling (1 : 40 000), the samples were mounted with mounting medium. Images were observed and photographed with fluorescence microscopy.

### 2.13 Statistical analysis

Quantitative variables are expressed as medians (interquartile ranges [IQRs]) or means (standard deviations, SD), and qualitative variables are expressed as absolute values (percentages). The Mann–Whitney *U* test was used to compare NK cytotoxicity and ligand expression between PCa patients and healthy participants. Paired *t* tests were used to compare preoperative and postoperative levels of ligand expression, CD63 expression, and cytotoxicity. Data shown in the bar graph are presented as the mean ± SD. Statistical analyses were performed using spss software version 22 (IBM, Armonk, NY, USA), with a two‐sided significance level set at *P* < 0.05.

### 2.14 Size exclusion chromatography

Size exclusion chromatography was purchased from IZON Science (Christchurch, New Zealand), and 1 mL of plasma was used to extract EVs following the manufactory protocol. Fraction 2 and 3 (700 μL/fraction) were collected and analyzed by NTA, and the protein concentrations of these two fractions were analyzed by BCA. For the SEC‐EVs function on NK cells, 10 μg·mL^−1^ EVs were used to treat NK‐92 cells (5 × 10^4^) for 24 h. DiI‐labeled K562 (1 × 10^5^) were co‐incubated with NK cells for 2 h, and the remaining DiI‐labeled K562 was measured by flow cytometry to examine the cytotoxicity of NK cells.

## 3 Results

### 3.1 Patient characteristics

In this cohort, the median (IQR) age of the patients was 65.38 (61.16–68.08) years. The median preoperative prostate‐specific antigen (IQR) level was 8.72 (6.17–13.63) ng·mL^−1^, and the mean C‐reactive protein level was 0.08 (0.04–0.13) mg·dL^−1^. Postoperative tumor pathology revealed that 16 patients had extracapsular extension of the prostate, 13 patients had seminal vesicle invasion, and six patients had pelvic lymph node metastasis. The numbers of patients classified as TNM stages II, III, and IV were 38 (48.1%), 30 (38.0%), and 6 (5.6%), respectively. Most patients had Grade 2 (42 patients, 53.2%) and Grade 3 (15 patients, 19.0%) Gleason scores. The median (IQR) NKA was 411.86 pg·mL^−1^ (156–895.6) preoperatively and 877.57 pg·mL^−1^ (431.63–2000) postoperatively. The number of NK cells was 7.24% (3.91–11.64) preoperatively and 7.82% (4.78–13.67) postoperatively (Table S1).

### 3.2 Prostate tumor removal altered NK‐cell characteristics

**Fig. 1:**
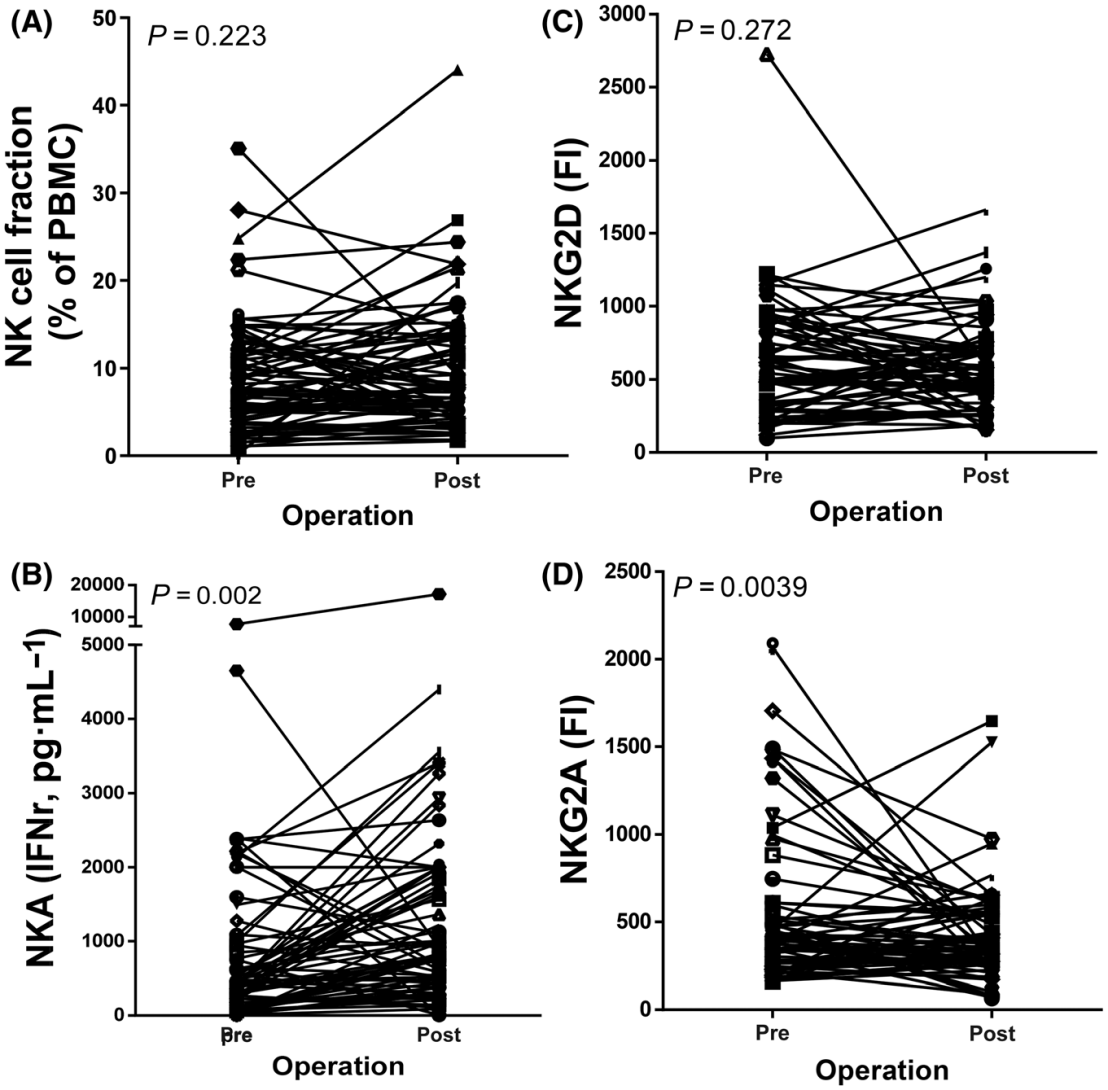
Removal of PCa altered NKA and NKG2A expression on NK cells. PBMCs were extracted from the blood of PCa patients before and 1 month after prostatectomy and stained with antibodies for analysis. Every parameter was compared in each patient before (pre) and after (post) surgery. The NK‐cell fraction (A) as well as NKA (B), NKG2D (C), and NKG2A (D) levels on NK cells were determined using flow cytometry. (A–D) Paired *t*‐test.

The existence of tumors may decrease NK‐cell number and NK‐cell cytotoxicity [27]. However, the NK‐cell characteristics after prostatectomy are not understood. After prostate tumor removal, the alterations in NK‐cell fraction did not reach significance (Fig. 1A, *P* = 0.223), but NKA increased dramatically (Fig. 1B, *P* = 0.002). As the regulatory receptors on NK cells, the changes in NKG2D before and after prostatectomy did not reach significance (Fig. 1C, *P* = 0.272), whereas the NKG2A level decreased significantly postoperatively (Fig. 1D, *P* = 0.0039). The postoperative expression level of NKG2A on NK cells was significantly lower than that before prostatectomy (326.56 vs. 384.29 (FI; median), *P* = 0.039). Characteristic changes in NK cells in each stage of PCa are shown in Fig. S1B–E and Tables 1 and 2. The proportion of patients with an increased NK‐cell number and NKG2D upregulation after prostatectomy decreased with increasing stage, and the alterations in NKG2A showed the opposite trend (Table 1). Due to the various sample sizes in each stage, patients with stage I and II disease and patients with stage III and IV disease were grouped together to observe the changes in NK characteristics. The proportions of NK cells and NKA, NKG2D and NKG2D/NKG2A expression levels, which all showed an increasing trend after prostatectomy, were higher in the stage I + II group compared with the stage III + IV group. The alterations in NKG2A after surgery exhibited opposite trends compared with other characteristics (Table 2). Tumor removal not only increased NKA but also decreased the NKG2A. The alteration of NKG2A after prostatectomy might be associated with NKA.

**Table 1:** Proportions of NK cells, NKA, NKG2D, NKG2A, and NKG2D/NKG2A increased after prostatectomy in four different stages.

Upregulation after surgery (%)	Stage 1	Stage 2	Stage 3	Stage 4
NK‐cell number	66.7	61.9	53.3	40.0
NKA	66.7	90.9	53.3	20.0
NKG2D	50.0	50.0	30.8	25.0
NKG2A	0.0	38.9	46.2	50.0
NKG2D/NKD2A	100.0	50.0	46.2	50.0

**Table 2:** Proportions of NK cells, NKA, NKG2D, NKG2A, and NKG2D/NKG2A increased after prostatectomy in different stages.

Upregulation after surgery (%)	Stages 1 + 2	Stages 3 + 4
NK‐cell number	62.5	50.0
NKA	88.0	45.0
NKG2D	50.0	29.4
NKG2A	35.0	47.1
NKG2D/NKD2A	55.0	47.1

### 3.3 EV number and characteristics differed in various tumor stages

**Fig. 2:**
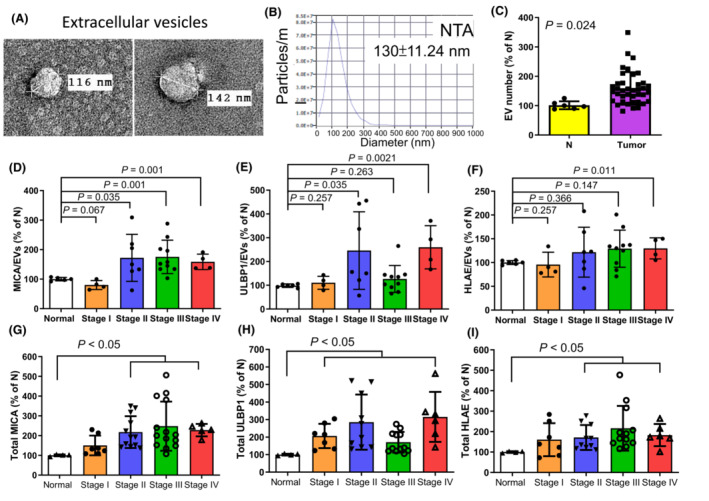
Extracellular vesicle number increased and the ligands for NKA regulatory receptors on EVs changed in the presence of tumors. (A) EVs were extracted from serum, and EV morphology was observed with TEM. (B) The size and number of EVs were measured by NTA. (C) A greater number of EVs was noted in tumor patients compared with healthy donors. The expression per EV (D–F) and total expression (G–I) of NKG2D ligands MICA and ULBP1 and the NKG2A ligand HLAE were analyzed based on tumor stages. (C) Paired *t* tests. (D–I) Mann–Whitney *U* test. Bar: mean ± SD.

Natural killer cell protein group 2D/A regulate NK cytotoxicity by binding with ligands on EVs in the tumor microenvironment [28]. However, the alteration and characteristics of circulating EVs among different stages of PCa are not understood to date. EVs were extracted from patient sera. EV morphology was observed under TEM, and the number of EVs was measured using NTA. TEM and NTA revealed that the average size of the EVs was approximately 130 nm, which was within the normal range (Fig. 2A,B). NTA was used to calculate the number of particles in each sample (Fig. 2B). The EV number in the blood was higher in patients compared with healthy donors (N) (Fig. 2C, *P* = 0.024). The interaction between ligands on EVs and receptors on NK cells has been studied across various cancer types [29, 30]. Cancer patients were stratified according to stage, but NK‐cell fraction, NKA, NKG2D, and NKG2A did not differ between patients of different stages (Fig. S2A–D). The levels of ligands for NKG2D (MICA and ULBP1) and NKG2A (HLAE) on each EV were significantly increased in patients with stage IV disease (*P* = 0.001, 0.0021, and 0.011, respectively) and MICA/EV levels also increased noticeably in patients with stage II and III disease (*P* = 0.035 and 0.001, respectively; Fig. 2D–F). In addition, the total expression levels of MICA and HLAE were increased in patients with stage II‐IV disease, and the total ULBP1 level was increased in all disease stages compared with healthy donors (*P* < 0.05; Fig. 2G–I). The alteration of ligands on EVs may be a regulator of NKA.

### 3.4 The EV fraction in the peripheral blood was altered after prostatectomy

**Fig. 3:**
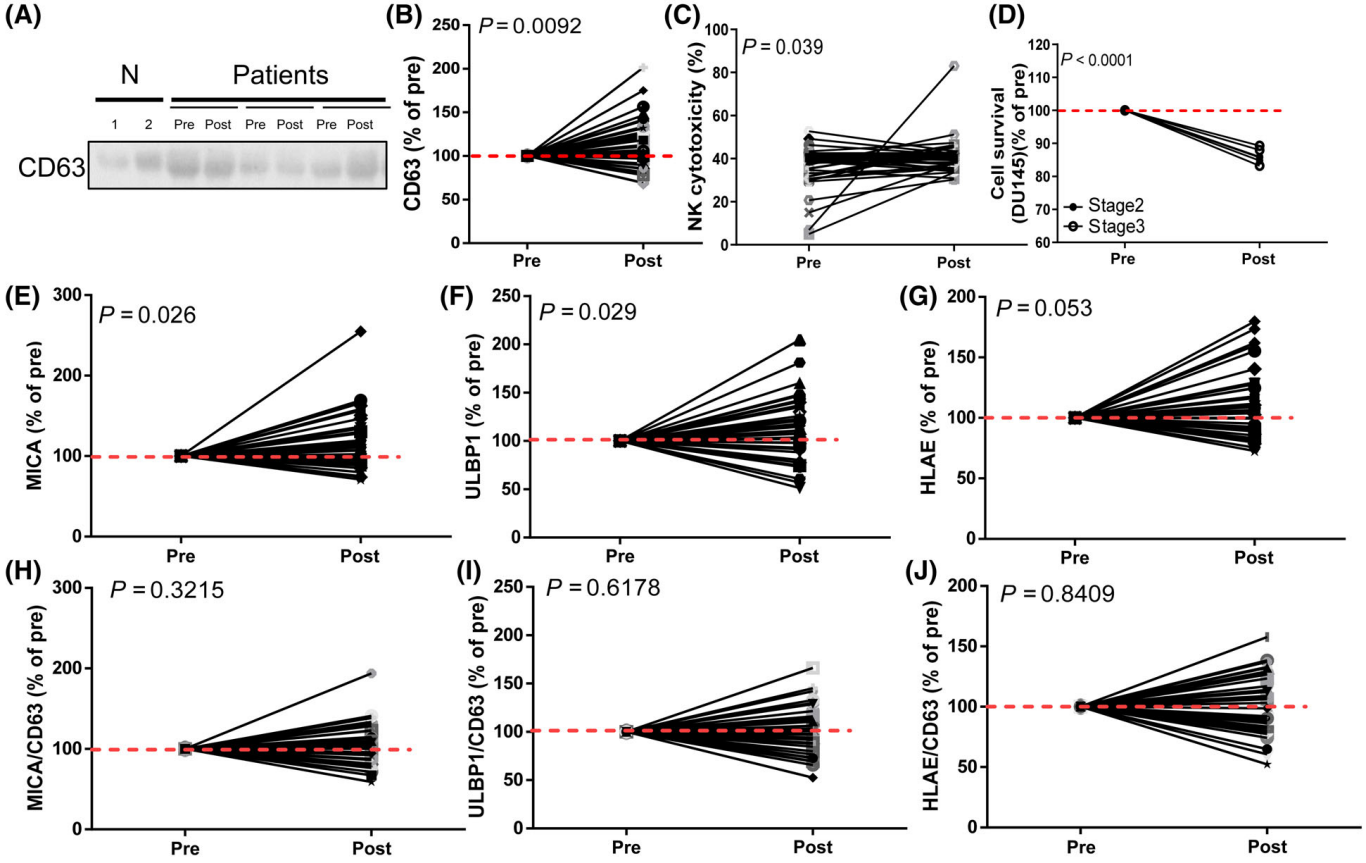
Extracellular vesicle number and ligands for NKGAD on EVs increased after PCa removal. (A, B) The EV number in patients before and after prostatectomy was measured based on CD63 expression, and the CD63 expression level was significantly altered in patients after prostatectomy. (C) Compared with EVs obtained from patients before the removal of PCa (pre), the EVs extracted from patients after prostatectomy (post) exhibited dramatically increased NK cytotoxicity. (D) The cell survival of DU145 under treatment of EVs‐treated NK92 cells. (E–J) Total expression levels of NKG2D ligands MICA (E) and ULBP1 (F) and NKG2A ligand HLAE (G) on EVs were analyzed before (pre) and after (post) prostatectomy. The expression levels per EV of MICA (H), ULBP1 (I), and HLAE (J) were also analyzed before (pre) and after (post) prostatectomy. The expression levels of ligands on EVs in the patients before prostatectomy (pre) were set as 100%. Red dashed line shows the level of 100%. (B–J) Paired *t* tests.

Extracellular vesicles in human blood carry various signals throughout the body [31]. However, whether the removal of tumor cells changes the composition or characteristics of EVs in the blood is unknown, and the change in EV number before and after prostatectomy was explored. The EV number was increased after prostatectomy (Post), as determined by NTA (Fig. S2E). The change in EV number was correlated with alterations of CD63 levels, which is an EV marker (Fig. S2F). CD63 levels were also positively correlated with CD9 levels, which is another EV marker (Fig. S2G). Therefore, the CD63 level was recognized to represent the number of EVs. Thirty‐nine pairs of EV samples were extracted from the blood before and after surgery, and CD63 expression levels were analyzed (Fig. 3A). After tumor removal (postoperation), CD63 levels were increased, indicating increased EV levels in the peripheral blood (Fig. 3B, *P* = 0.0092, and Fig. S2H).

### 3.5 The ligands on EVs changed after tumor removal

After treatment with pre or postoperation EVs, NK cytotoxicity on K562 cells was significantly increased after tumor removal (32 of 40 were increased, *P* = 0.039; Fig. 3C). Also, the NK cytotoxicity on DU145 PCa cells was also increased in postoperation EVs‐treated NK compared with the preoperation EVs‐treated NK (*P* < 0.0001; Fig. 3D, solid circle: EVs from stage 2 patients, open circle: EVs from stage 3 patients). Given that NK activity is tightly correlated with ligands on EVs [32], expression levels of ligands on EVs were evaluated (Fig. S2I). Total ligand expression and the ligands present on each EV were compared before and after prostatectomy. The expression of ligands in patients with prostatectomy (post) was normalized to that in patients before operation (pre). The overall MICA and ULBP1 expression levels, which are ligands for NKG2D, were significantly increased after surgery compared with that noted before surgery (*P* = 0.026 and 0.029, respectively), but the alteration in HLAE levels did not reach significance (*P* = 0.053; Fig. 3E–G). The changes in all ligands per EV (ligands/CD63) did not reach significance (0.3215, 0.6178, and 0.8409, respectively; Fig. 3H–J). Therefore, the increased ligand expression on EVs might be associated with the increased NKA after prostatectomy.

### 3.6 NKG2A is critical for NKA alterations in PCa patients

**Fig. 4:**
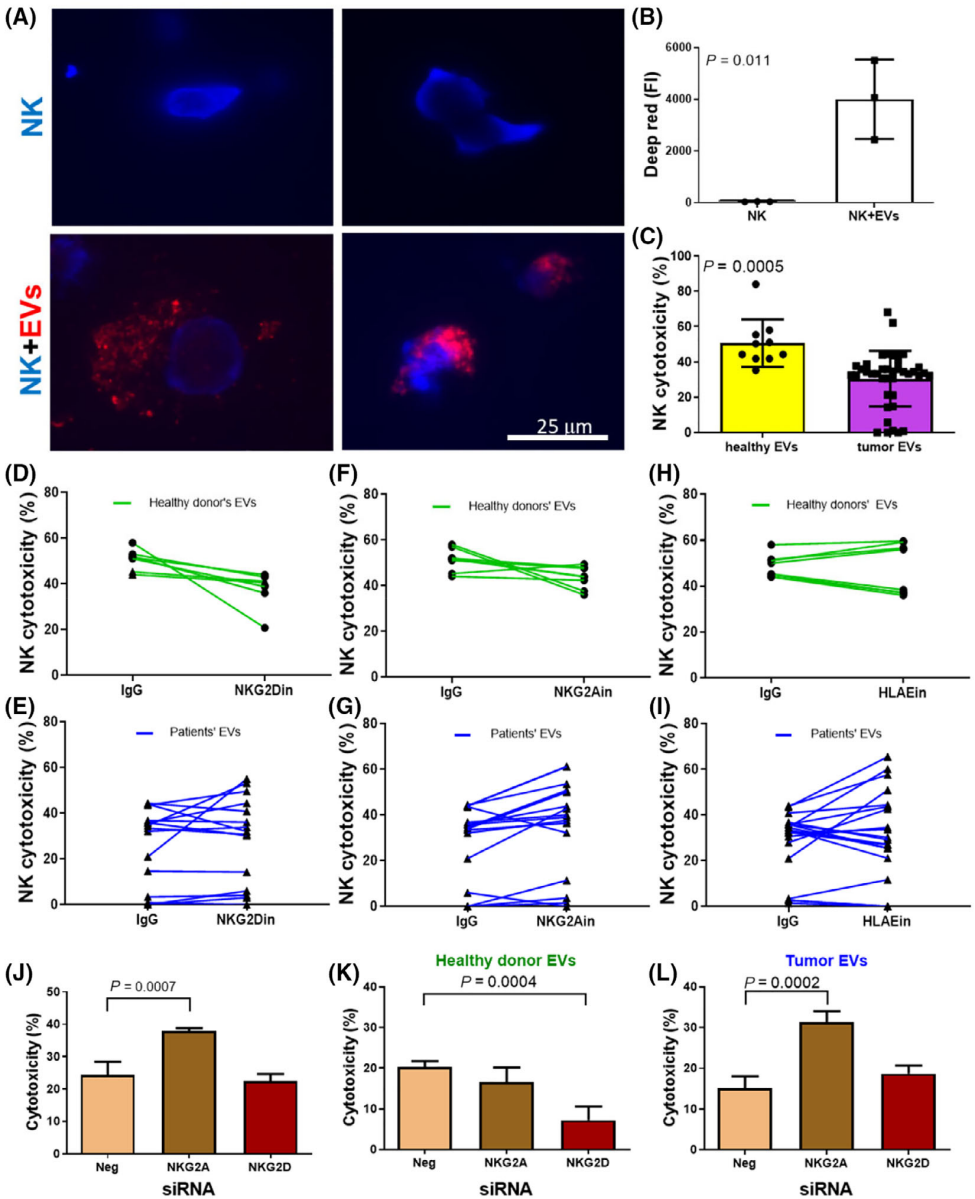
Extracellular vesicles interacted with NK cells, and NKG2A was the key regulator of NK cytotoxicity in tumor patients. (A) EVs labeled with deep red fluorescence were incubated with NK92 cells for 24 h. Cells were observed under fluorescence microscopy. The cell nuclei of NK92 cells were labeled with Hoechst 33342. EVs were attached to NK92 cells or located in the cytoplasm of NK cells. (B) Deep red fluorescence inside NK cells was analyzed using flow cytometry. The deep red FI was significantly increased in NK cells + EVs compared with NK92 cells alone. (C–I) Equal amounts of EVs were added to NK92 cells and incubated for 24 h. The cytotoxic effects of NK cells toward K562 cells, which were labeled with CM‐DiI, were measured using flow cytometry. (C) EVs extracted from patients promoted significantly lower NK cytotoxicity than those extracted from healthy donors. (D, E) NKG2D inhibitory antibody decreased NK cytotoxicity in healthy donors (8 of 8), whereas NK cytotoxicity was only decreased in half of the patients (7 of 15). (F, G) NKG2A inhibitory antibody increased NK cytotoxicity in patients (13 of 15) but only in a few of the healthy donors (1 of 8). (H, I) HLAE increased NK cytotoxicity in half of the healthy donors (4 of 8) and PCa patients (10 of 20). (J–L) NK cytotoxicity in cells with siRNAs targeting nothing (Neg), NKG2A and NKG2D was measured with treatment of medium only (J) and EVs from healthy donors (K) and tumor patients (L). (B, C) Paired *t* tests. (J–L) Mann–Whitney *U* test. Scale bar: 25 μm. Bar: mean ± SD.

To further investigate which receptor on NK cells is the key regulator of NKA, an *in vitro* study of NK92 cells was performed. We first determined whether EVs extracted from sera bound to NK92 cells. EVs were labeled with deep red and incubated with NK92 cells for 24 h. Images showed that EVs bound to the surface and were also present inside NK cells (Fig. 4A). The deep red FI was significantly increased in the NK + EV group compared with NK cells alone as assessed using flow cytometry analysis (Fig. 4B; Fig. S2J). NK‐cell cytotoxicity was analyzed by adding EVs from healthy donors or tumor patients. NK cytotoxicity was dramatically decreased by EVs from tumor patients (Fig. 4C, *P* = 0.0005). To determine the main regulatory receptor for NKA, specific inhibitory antibodies and siRNAs against receptors and ligands were used. Administration of the NKG2D inhibitory antibody decreased NK cytotoxicity in all eight healthy donors but decreased cytotoxicity in only half of the tumor patients (7 of 15 patients; Fig. 4D,E). In contrast, addition of the NKG2A inhibitory antibody increased NK cytotoxicity in 86.7% of cancer patients (13 of 15 patients) but in only 12.5% of healthy donors (1 of 8; Fig. 4F,G). The inhibitory antibody against HLAE, which is the ligand for NKG2A, increased NK cytotoxicity in half of healthy participants and cancer patients (Fig. 4H,I). Regarding siRNAs targeting NKG2A or NKG2D, siNKG2A significantly increased cytotoxicity in NK cells (Fig. 4J, *P* = 0.0007). When treated with circulating EVs from healthy donors, siNKG2D significantly decreased NK cytotoxicity (Fig. 4K, *P* = 0.0004), whereas siNKG2A dramatically increased cytotoxicity in NK cells treated with circulating EVs from tumor patients (Fig. 4L, *P* = 0.0002). These results indicated that NKG2A was the main regulator of NKA in tumor patients.

### 3.7 The infiltration of NK cells in tumors and adjacent normal regions was altered in different stages

**Fig. 5:**
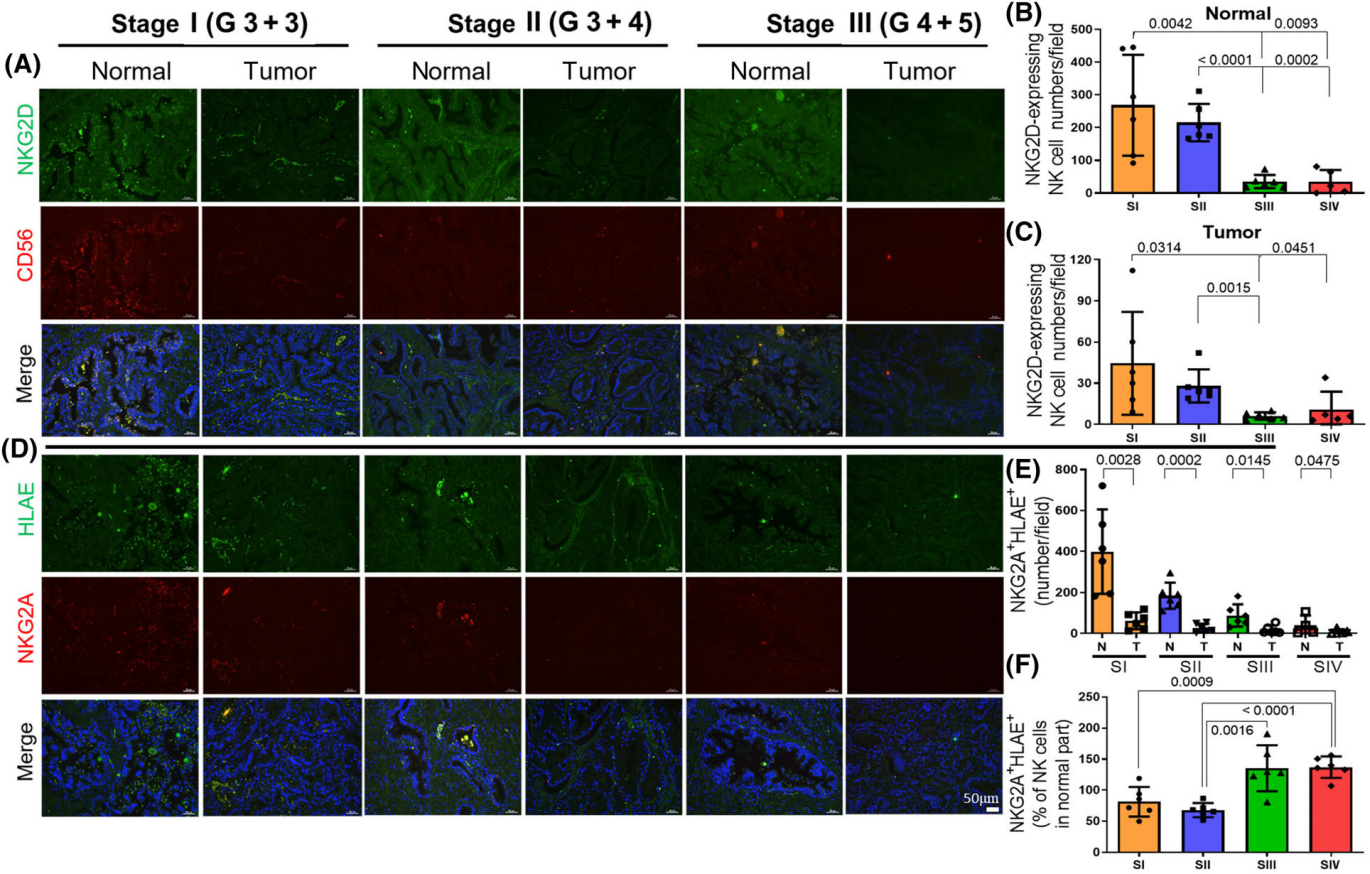
Natural killer‐cell infiltration and the binding of HLAE and NKG2A in PCas and adjacent normal tissues varied at different stages. (A) Prostate tumor tissues from PCa patients at various stages were stained with NKG2D (green), CD56 (red), and DAPI (blue). NK cells with the colocalization of NKG2D and CD56 are indicated in yellow. The NK‐cell number was decreased in adjacent normal (B) and tumor regions (C) in higher stages. (D) Tissues were stained with HLAE (green), NKG2A (red), and DAPI (blue). The binding of HLAE and NKG2A is indicated in yellow, and the colocalization of green and red fluorescence in adjacent normal (N) and tumor tissues (T) is shown (E). (F) The colocalization of NKG2A and HLAE inside NK cells in adjacent normal tissues was calculated. Scale bar: 50 μm. G, Gleason Score; SI, stage 1; SII, stage 2; SIII, stage 3; SIV, stage 4. (B–F) Mann–Whitney *U* test. Bar: mean ± SD.

The results reported above indicate that NKG2A serves as the key regulator of NKA in tumor patients; however, whether NKG2A is inhibited by ligands in tumor patients is unclear. IHC was performed to investigate the infiltration of NK cells in tumors and adjacent normal tissues. The number of infiltrated NK cells, which were labeled by NKG2D (green) and CD56 (red) and shown in yellow in the merged panel, was lower in tumor tissue than normal tissue in every stage. The number of NKG2D‐expressing NK cells was lower in the normal region in stage III and IV patients compared with stage I and II patients. The alterations in NK numbers in the adjacent normal region were similar to that observed in the tumor region, but the number of infiltrated of NK cells in the tumor region was reduced compared with that noted in the normal tissues (Fig. 5A–C). NKG2A and HLAE were labeled to examine the interaction between EVs and NK cells (Fig. 4D). NKG2A and HLAE fluorescence levels were also lower in the tumor tissue than in the adjacent normal tissue (Fig. 4E). Increased colocalization of NKG2A and HLAE (yellow fluorescence) was noted in the normal region among those labeled with NKG2D and CD56 in patients with stage III and IV disease compared with those with stage I and II disease (Fig. 4F). These findings indicated the binding of NK cells and EVs.

## 4 Discussion

Extracellular vesicles in body fluids are stable and may serve as disease markers [33]. Tumor‐derived EVs in the peripheral blood could represent an important element for regulating NKA [34, 35]. However, the EV composition in the peripheral blood should function as a better marker for recognizing the status of the tumor microenvironment [10]. Based on a cohort of PCa patients at National Taiwan University, more circulating EVs were presented in the peripheral blood in tumor patients than in healthy donors (Fig. 2); however, the EV number was not stage dependent (Fig. S1B, *P* = 0.056). The levels of the total ligands MICA, ULBP1, and HLAE were increased in more advanced tumor patients (Fig. 2). The increased levels of MICA and ULBP1 bound to NKG2D on NK cells and could lead to NK‐cell exhaustion before the appearance of tumor cells [36], and the increase in HLAE levels inhibited NKA by binding to NKG2A [37]. Therefore, the screening of total ligand expression on circulating EVs may serve as a marker for evaluating the presence of tumors.

Although studies have indicated that EVs may regulate NKA through binding to receptors on NK cells [38], whether receptors change after prostatectomy is not understood. After tumor removal, NKA levels increased, and the level of the inhibitory receptor NKG2A was significantly reduced. In contrast, the NK‐cell number and the level of the activating receptor NKG2D were not significantly altered (Fig. 1; Tables 1 and 2). In addition, the number of circulating EVs number and NKA were significantly increased after surgery compared with before surgery (Fig. 3C,D). The increased number of EVs may be derived from normal cells and contain information that helps tissue recovery from surgery [39, 40]. These results indicated that NKG2A and circulating EVs are potentially correlated and important for NKA after prostatectomy.

Natural killer cell activity was reduced in patients with advanced‐stage disease [41]. NKA is assessed based on the release of interferon production regulator (IFNr), perforin, or granzyme B. The present study used NKVue to examine the IFNr level released from NK cells, and NKVue levels positively correlated with perforin levels (Fig. S2K). Circulating EVs from tumor patients led to decreased NKA (Fig. 4C). The binding of receptors on the NK membrane with ligands on EVs finely regulates NKA [38]. Although NKG2A was found to be important for NKA, the alteration in the total expression levels of the NKG2A ligand HLAE did not reach significance after tumor removal (Fig. 3G). Instead, the levels of NKG2D ligands MICA and ULBP1 were significantly increased (Fig. 3E,F) along with the increase in EV number (Fig. 3A,B). Therefore, whether NKG2A or NKG2D is the key regulator of NKA needs to be determined. The importance of receptors on NK cells was evaluated by administering inhibitory antibodies or siRNAs. The addition of a NKG2D inhibitory antibody decreased NK cytotoxicity in only half of patients (7/15) in contrast to that observed in healthy donors (8/8), and 86% (13/15) of NK cytotoxicity was increased in patients with the addition of NKG2A inhibitory antibody (Fig. 4D–G). The siRNAs targeting NKG2D and NKG2A showed a trend similar to that of inhibitory antibodies (Fig. 4J–L). These results indicated that NKG2A was the dominant receptor for regulating NKA in tumor patients, and NKG2A blockade may be critical for maintaining NK activity against tumors. Evidence in the literature also indicates that disruption of NKG2A, such as that obtained with monalizumab [42], induces effective antitumor immunity, but the clinical efficacy of NKG2A inhibitory antibodies is unclear [43]. As the major ligand of NKG2A on EVs, HLAE is overexpressed in several tumors [44] and is correlated with tumor prognosis [44]. The HLAE inhibitory antibody only affected NK cytotoxicity by 50% (Fig. 4H,I) in both healthy and tumor patients. This finding suggests the existence of other ligands for NKG2A in PCa, although it is the major ligand for NKG2A at present.

Furthermore, NKA increased dramatically after prostatectomy, but NK‐cell number was not altered. As the average life span of NK cells is approximately 2 weeks [45], NK cells in the blood are newly developed in patients 1 month after surgery. Therefore, given the increase in EV number (Fig. 3B), the increase in NKA (Figs 1B and 3C,D) after surgery could result from the increase in new NK cells with increased NKG2D ligands on circulating EVs. Combined with the decrease in NKG2A on NK cells and the increase in NKG2D ligand on circulating EVs, NKA could be increased after tumor removal.

Immunostaining of NK cells (CD56^+^/NKG2D^+^) and the interaction of NKG2A and HLAE were assessed in tumor and adjacent normal tissues to validate the results found in the *in vitro* model. NK cells were identified by the colocalization of NKG2D and CD56, which are shown as yellow spots in the merged panel. Fewer NK cells infiltrated in the tumor tissue compared with that noted in the adjacent normal tissue in patients at every stage. The number of infiltrating NK cells was lower in patients with higher grade PCa in both the tumor tissue and adjacent normal tissue (Fig. 5A–C). This finding was consistent with those of other studies that demonstrated an association of a high density of tumor‐infiltrating NK cells with a better prognosis in multiple human solid tumors [46, 47]. Although there were fewer NK cells inside the tumor tissue, the binding of HLAE and the NKG2A receptor was still observed as yellow spots. More yellow spots were noted in the adjacent normal tissues in patients with higher stage disease than in patients with lower stage disease (Fig. 5D–F). These results suggested that NK cells have difficulty infiltrating into tumor tissue, and tumor cells may release EVs to regulate NKA through binding to NKG2A on NK cells.

The EV extraction method used in this study was the polyethylene glycol (PEG)‐based method. There are numerous EV extraction methods developed recently [48, 49]. In order to further confirm the EVs' function, SEC method was also used to extract EVs (Fig. S3A). The sizes and functions of EVs were measured by NTA and NK cytotoxicity tests (Fig. S3B–D). The sizes and functions of SEC‐EVs were similar with PEG‐EVs. These results indicated the circulating EVs extracted by either PEG or SEC method from PC patients can regulate NK function.

## 5 Conclusion

**Fig. 6:**
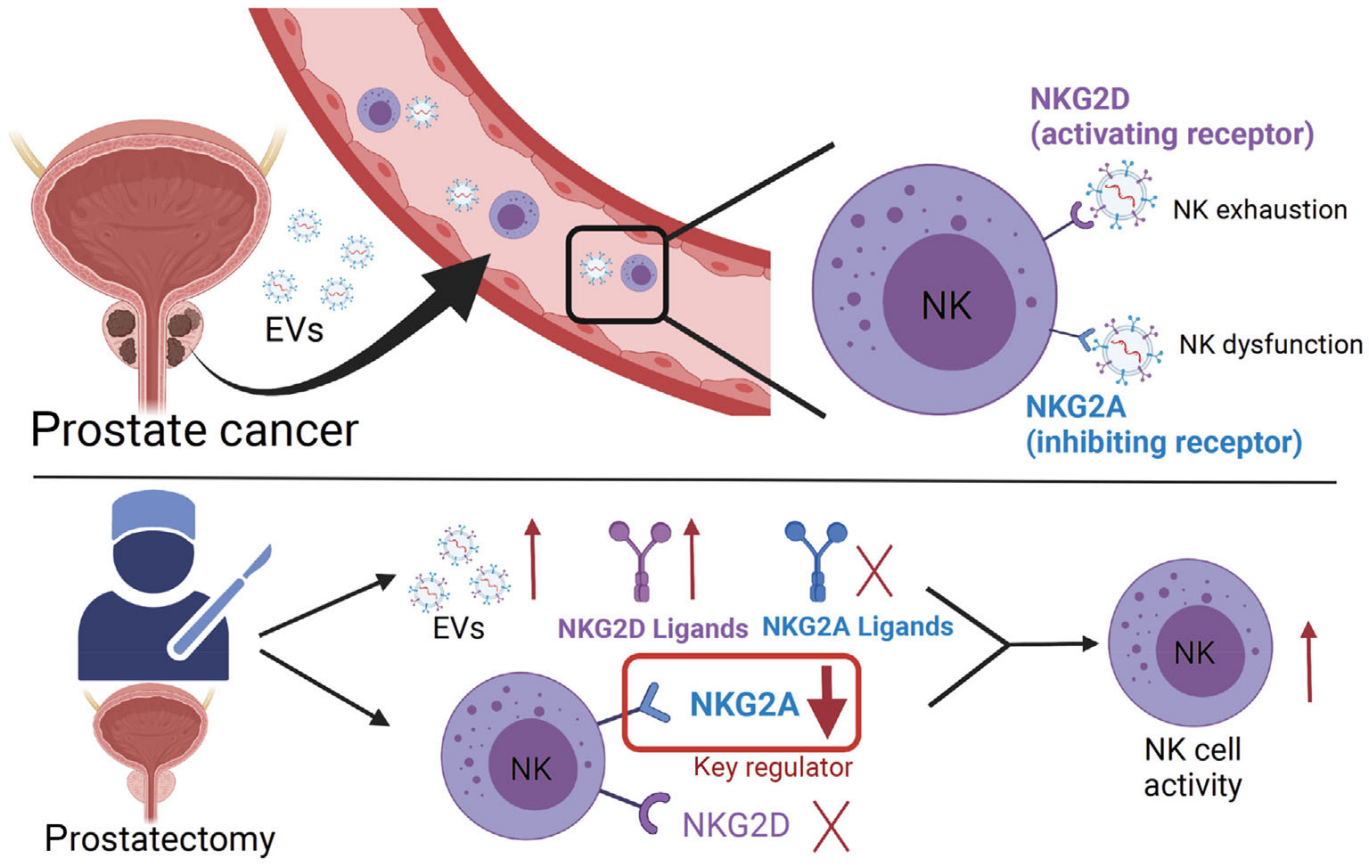
Schematic diagram of this study. More circulating EVs were found in the blood of prostate tumor patients. Two types of receptors mainly regulate NKA: NKG2D, the activating receptor that leads to NK activation or exhaustion, and NKG2A, the inhibiting receptor that leads to NK dysfunction. After treatment with an inhibitory antibody or siRNA, NKG2A was shown to be the main receptor regulating NKA through EV binding. After prostatectomy, the number of circulating EVs increased, which subsequently increased NKA by decreasing NKG2A and increasing total NKG2D ligands. In conclusion, NKG2A is the key regulator of NKA in PCas, and blocking NKG2A could represent a potential therapy for inhibiting the effects of tumor‐dependent EVs on NKA.

In conclusion, with the characteristic alteration of circulating EVs, NKA, which was reduced in PCa patients, was restored after prostatectomy. The increased NKA after prostatectomy may result from the decrease in NKG2A levels on NK cells and the increased NKG2D ligands on circulating EVs (Fig. 6). Notably, NKG2A is the main regulator of NKA in PCa before and after prostatectomy, and the development of methods to prevent EV‐associated NKG2A inhibition may enhance the efficacy of immunotherapy in PCa patients.

## Conflict of interest

Nonfinancial support from ATGen was received during the performance of the study. The NKVue kit was provided by ATGen, which did not participate in the study design or statistical analyses and was blinded to the results.

## Author contributions

Y‐CL, C‐HH, and H‐NK conceived of the present idea. Y‐CL, C‐HH, J‐HH, Y‐AL, and M‐CK collected the clinical samples. J‐AL, C‐HC, AH, and H‐NK performed the experiments. F‐SJ, JC‐HC, C‐YH, K‐PC, and C‐HC verified the analytical methods. Y‐CL and H‐NK supervised the findings of this work. Y‐CL and H‐NK wrote the manuscript with supports from Y‐AL, F‐SJ, JC‐HC, C‐YH, and K‐PC. All authors discussed the results and contributed to the final manuscript.

### Peer Review

The peer review history for this article is available at https://www.webofscience.com/api/gateway/wos/peer‐review/10.1002/1878‐0261.13422.

## Supporting information


**Fig. S1.** Gating strategy for NK cells and various parameters of NK in different stages of prostate cancer patients. (A) The gating strategy for NK cells (CD3^−^/CD56^+^/CD16^+^). (B‐E) The NK number, NKA, NKG2D, and NKG2A in patients with different stages of prostate cancer patients. NK: natural killer cell, NKA: natural killer cell activity.
**Fig. S2.** Various parameters for NK and EV analysis in different stages of prostate cancer patients, including NK fraction, NKA, NKG2D, NKG2A of NK, ligands on Evs. (A‐D) The comparison of NK‐cell fraction, NKA, NKG2D, and NKG2A in patients before (pre) or after (post) prostatectomy with different stages (indicated with different colors). (E) The EV number measured by NTA. (F) The expression of CD63 on EVs. (G) The correlation of CD63 and CD9 levels in EVs. (H) The CD63 levels in circulating EVs in patients before (pre) or after (post) prostatectomy with different stages. (I) The expressions of ligands on EVs. (J) The deep red level inside NK cells. (K) The correlation of NKVue (IFNr) and perforin. EV: extracellular vesicle, NK: natural killer cell, NKA: natural killer cell activity, IFNr: interferon r.
**Fig. S3.** Results of EV with polyethylene glycol (PEG)‐based method was verified with EVs with size exclusion chromatography (SEC) method. (A) The SEC column used in the EV extraction. (B) The sized of EVs in the fraction 2 and 3 analyzed with NTA. (CD) The remaining K562 (NK cytotoxicity test) of NK cells treated with SEC‐EVs from healthy donors, PC patients before (pre‐) and after (post‐) prostatectomy. EV: extracellular vesicle, NK: natural killer cell.

**Table S1.** Basic information of prostate cancer patients included in this study.

## Data Availability

All data are available on request.
