# Supplementary figures and images for: NKG2A and circulating extracellular vesicles are key regulators of natural killer cell activity in prostate cancer after prostatectomy
Source: Mol Oncol. 2023 Mar 27;17(8):1613–27. doi: 10.1002/1878-0261.13422 (PMC10399716; doi:10.1002/1878-0261.13422)

Supplementary figure 1

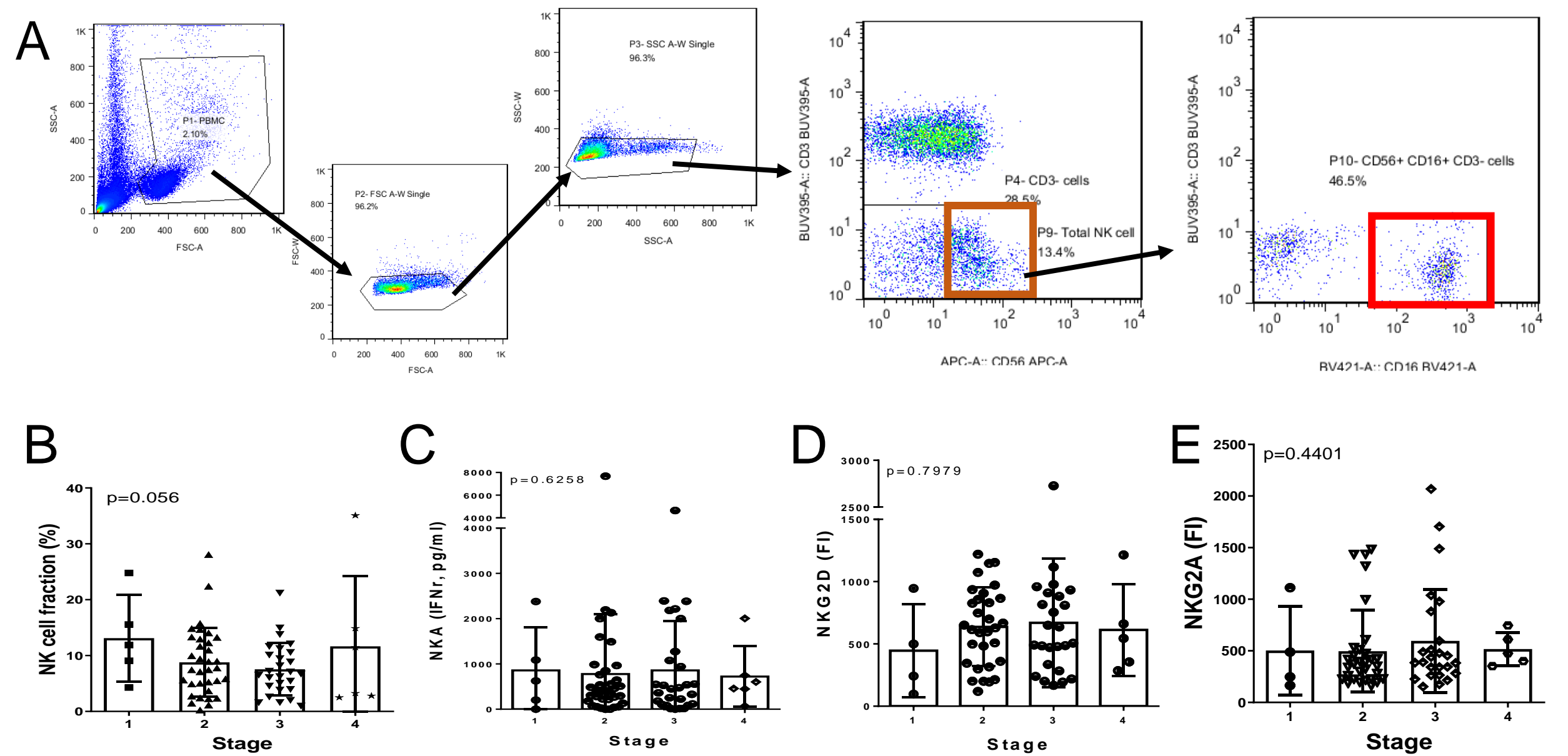

Supplementary figure 2

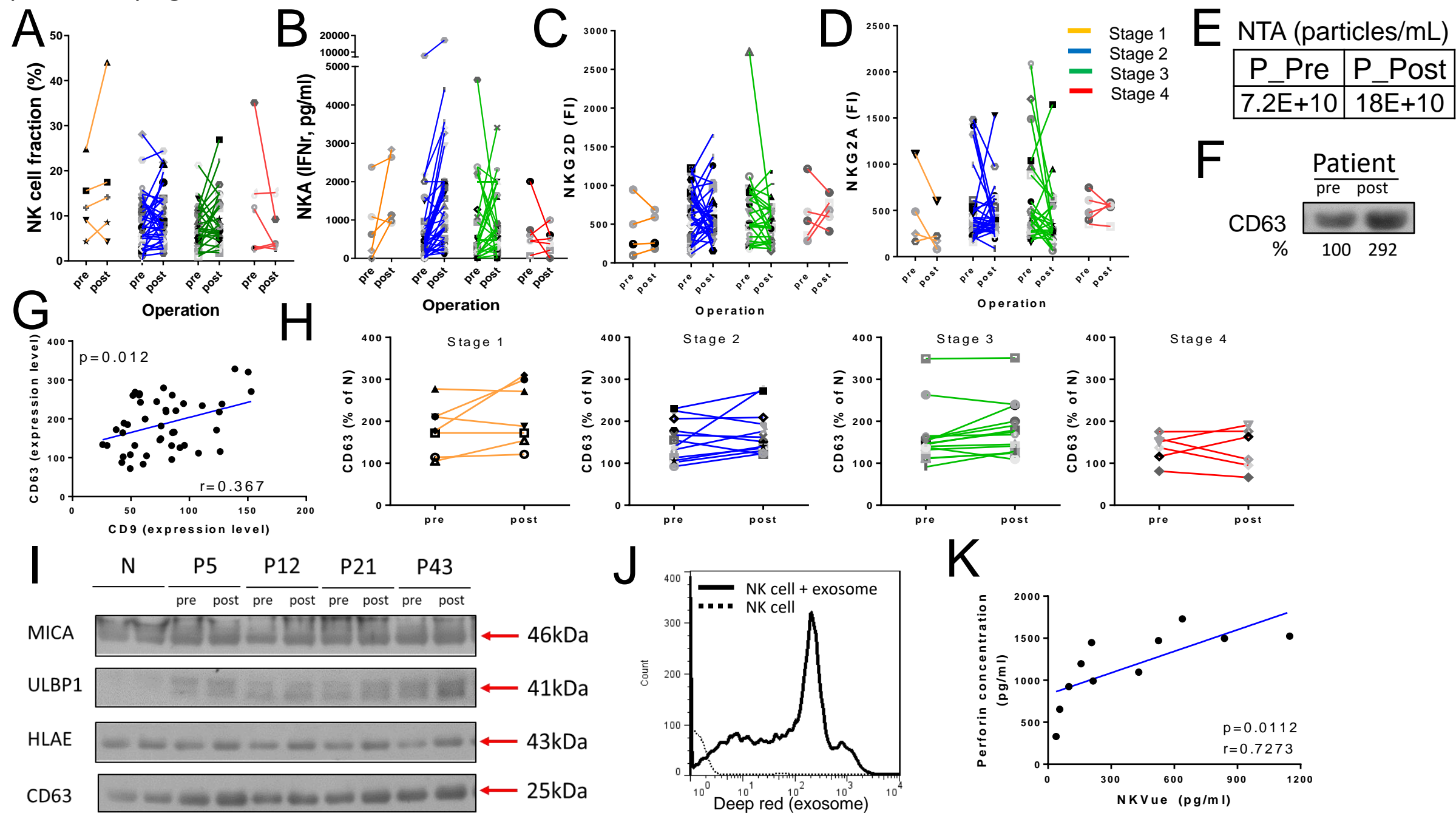

Supplementary figure 3

A

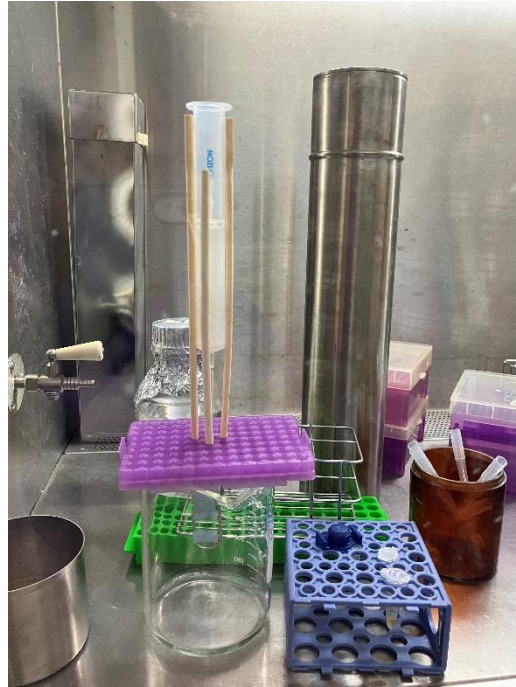

B

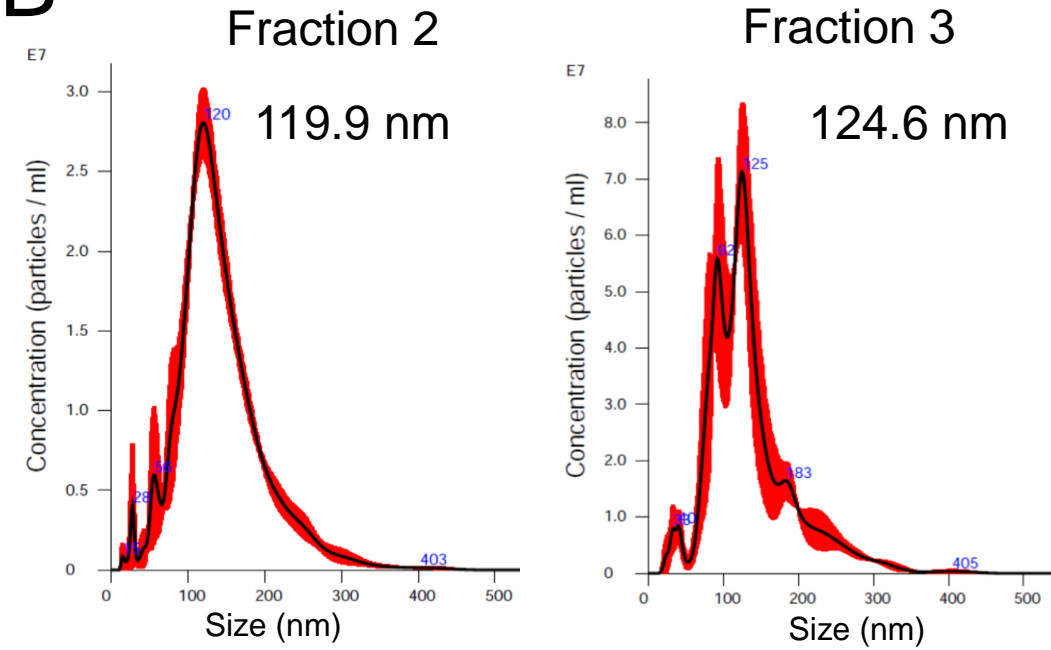

C

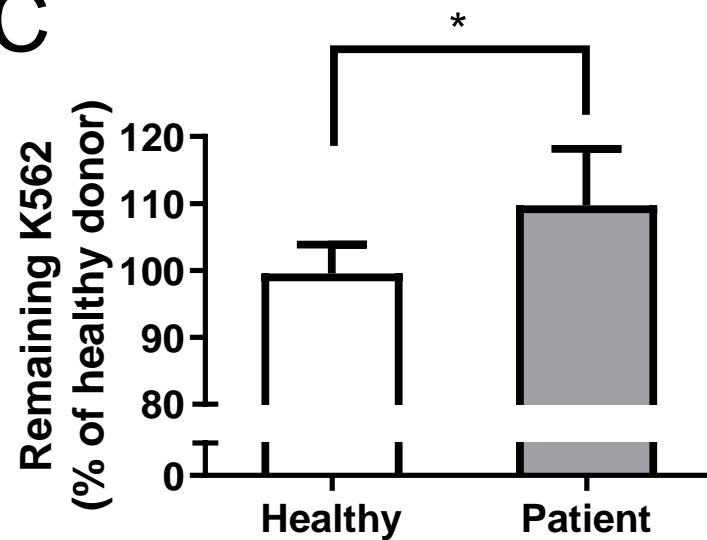

D

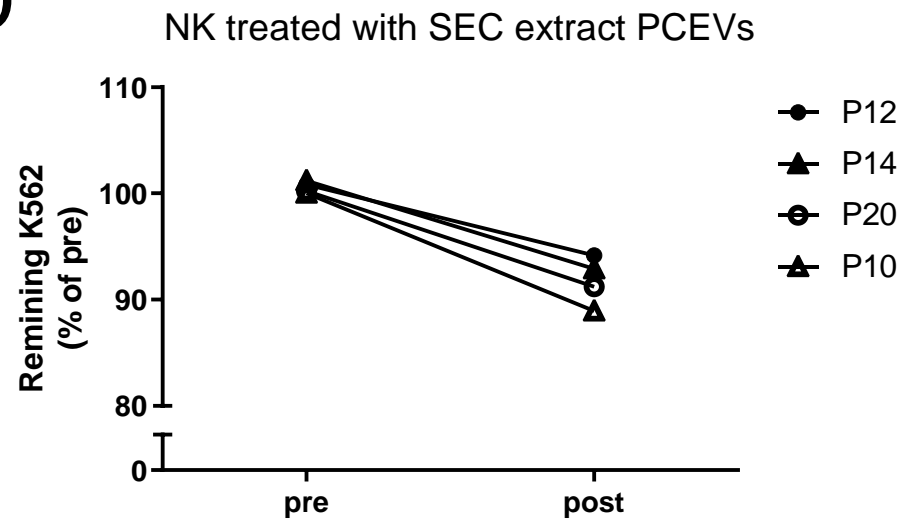

Supplement: Supplementary file 1 — Fig. S1. Gating strategy for NK cells and various parameters of NK in different stages of prostate cancer patients. (A) The gating strategy for NK cells (CD3−/CD56+/CD16+). (B‐E) The NK number, NKA, NKG2D, and NKG2A in patients with different stages of prostate cancer patients. NK: natural killer cell, NKA: natural killer cell activity. Fig. S2. Various parameters for NK and EV analysis in different stages of prostate cancer patients, including NK fraction, NKA, NKG2D, NKG2A of NK, ligands on Evs. (A‐D) The comparison of NK‐cell fraction, NKA, NKG2D, and NKG2A in patients before (pre) or after (post) prostatectomy with different stages (indicated with different colors). (E) The EV number measured by NTA. (F) The expression of CD63 on EVs. (G) The correlation of CD63 and CD9 levels in EVs. (H) The CD63 levels in circulating EVs in patients before (pre) or after (post) prostatectomy with different stages. (I) The expressions of ligands on EVs. (J) The deep red level inside NK cells. (K) The correlation of NKVue (IFNr) and perforin. EV: extracellular vesicle, NK: natural killer cell, NKA: natural killer cell activity, IFNr: interferon r. Fig. S3. Results of EV with polyethylene glycol (PEG)‐based method was verified with EVs with size exclusion chromatography (SEC) method. (A) The SEC column used in the EV extraction. (B) The sized of EVs in the fraction 2 and 3 analyzed with NTA. (CD) The remaining K562 (NK cytotoxicity test) of NK cells treated with SEC‐EVs from healthy donors, PC patients before (pre‐) and after (post‐) prostatectomy. EV: extracellular vesicle, NK: natural killer cell. [file MOL2-17-1613-s001.pdf]
