# Supplementary material for: NKG2A and circulating extracellular vesicles are key regulators of natural killer cell activity in prostate cancer after prostatectomy
Source: Mol Oncol. 2023 Mar 27;17(8):1613–27. doi: 10.1002/1878-0261.13422 (PMC10399716; doi:10.1002/1878-0261.13422)
Supplement: Supplementary file 2 — Table S1. Basic information of prostate cancer patients included in this study. [file MOL2-17-1613-s002.docx]

**Supplementary Table 1**

| **Basic patient information** | |
| --- | --- |
| Number of patients | 79 |
| Age | 65.38 (61.16-68.08) |
| Preoperative PSA (ng/mL) | 8.72 (6.17-13.63) |
| Prostate volume (ml) | 30.30 (22.58-38.58) |
| White blood cells count (cells/mL) | 6.20 (5.535-7.02) |
| C-Reactive protein (mg/dL) | 0.08 (0.04-0.13) |
| Tumor characteristics No. (%) | |
| Extracapsular extension of prostate | 16 (20.3) |
| Seminal vesicle invasion | 13 (16.5) |
| Pelvic lymph node metastasis | 6 (7.6) |
| TNM staging, No. (%) | |
| I | 5 (6.3) |
| II | 38 (48.1) |
| III | 30 (38.0) |
| IV | 6 (7.6) |
| Gleason score, No. (%) | |
| Grade Group 1 [≤6] | 8 (10.1) |
| Grade Group 2 [7(3+4)] | 42 (53.2) |
| Grade Group 3 [7(4+3)] | 15 (19.0) |
| Grade Group 4 [8] | 4 (5.1) |
| Grade Group 5 [9–10] | 10 (12.7) |
| Pretreatment risk stratification | |
| Low | 18 (22.8) |
| Intermediate | 36 (45.6) |
| High | 18 (22.8) |
| Very high | 7 (8.9) |
| Preoperative NK cell activity NKVue, pg/mL | 411.86 (156-895.6) |
| Postoperative NK cell activity NKVue, pg/mL | 877.57 (431.63-2000) |
| Preoperative NK cell number (%) # | 7.24 (3.91-11.64) |
| Postoperative NK cell number (%) # | 7.82 (4.78-13.67) |

# The NK cell number was the percentage of NK in the PBMC.
